# Options for Regenerative Treatment with Bone Grafts in Children with Anterior Lip/Palate Cleft—A Review

**DOI:** 10.3390/children12050559

**Published:** 2025-04-26

**Authors:** Anna Miguel-Escribano, Cosimo Galletti, Juan de Quixano-Bardaji, Francisco Real-Voltas, Luca Fiorillo, Maria Cuevas-Nunez, Fulvia Galletti, Javier Flores-Fraile

**Affiliations:** 1 Department of Integrated Dentistry, School of Dentistry, Universitat Internacional de Catalunya, 08195 Sant Cugat del Vallés, Spain; anna.miguel@uic.es (A.M.-E.); juanchobdeg@uic.es (J.d.Q.-B.); freal@uic.es (F.R.-V.); 2 School of Medicine and Surgery Kore, University of Enna, 94100 Enna, Italy; cosimo.galletti@unikore.it; 3 Department of Dental Cell Research, Dr. DY Patil Dental College and Hospital, Dr. DY Patil Vidyapeeth, Pune 411018, Maharashtra, India; lfiorillo@unime.it; 4 Department of Biomedical and Dental Science and Morphological and Functional Imaging, University of Messina, 98166 Messina, Italy; mcuevasnunez@uic.es (M.C.-N.); fulviagalletti@gmail.com (F.G.); 5 Department of Surgery, Faculty of Medicine, University of Salamanca, 37007 Salamanca, Spain

**Keywords:** cleft lip, cleft palate, embryology, etiology, epidemiology, bone graft, alveolar bone grafting, secondary alveolar bone grafting

## Abstract

Anterior lip/palate cleft is a congenital deformity affecting the upper lip and palate, posing significant challenges in both aesthetic and functional aspects for children. Effective management of this condition is crucial for improving quality of life and ensuring normal development. This review aims to highlight the regenerative treatment options available for children with anterior lip/palate cleft, focusing on the use of bone grafts and other advanced dental procedures. A review of the current literature and clinical practices was conducted to identify and evaluate the most effective treatment options. Emphasis was placed on regenerative techniques, particularly the use of bone grafts. Advancements in regenerative dentistry offer promising outcomes for children with anterior lip/palate cleft. Bone grafts, combined with innovative techniques such as growth factors, stem cell therapy, and distraction osteogenesis, provide effective solutions for restoring function and aesthetics. A multidisciplinary approach is essential to ensure comprehensive care and optimal results for these patients.

## 1. Introduction

Anterior lip/palate cleft (ALPC) is a congenital anomaly characterized by the incomplete fusion of the upper lip, palate, and often the alveolar ridge during embryogenesis, typically between the 4th and 7th weeks of fetal development. This malformation results in an alveolar cleft, a bony defect in the maxillary arch that disrupts the continuity of the dental-bearing bone. The condition’s global prevalence varies widely, ranging from 1 in 500 to 2500 live births, influenced by geographic, ethnic, and socioeconomic factors [1]. For example, epidemiological data indicate a prevalence of 9.4 per 10,000 births in Colombia and 10.3 per 10,000 in Chile, while Native American populations exhibit rates as high as 20 to 35 per 10,000 births, highlighting significant ethnic disparities [1]. Beyond its physical manifestations, ALPC profoundly impacts multiple domains, including facial aesthetics, feeding efficiency, speech articulation, and dental alignment, necessitating a multidisciplinary approach to treatment [2]. Without timely intervention, these functional deficits can persist, complicating craniofacial development and psychosocial well-being.

The primary objective of this review is to assess regenerative treatment options for children with ALPC, with a particular emphasis on bone grafting strategies to address the alveolar cleft. These interventions aim to restore the structural integrity of the alveolar process, enabling proper eruption of permanent teeth, stabilizing the maxillary arch, and enhancing facial symmetry. Successful grafting also lays the foundation for advanced dental rehabilitation, such as osseointegrated dental implants, which are critical for long-term oral function and quality of life [3,4]. Historically, surgical correction of alveolar clefts (AC) has evolved from simple soft tissue closure to sophisticated regenerative techniques, reflecting advancements in biomaterials and molecular biology.

Autologous bone grafts, harvested from the patient’s own body, are considered the gold standard for AC repair due to their triad of osteogenic (bone-forming), osteoinductive (bone-stimulating), and osteoconductive (bone-supporting) properties [5,6]. The iliac crest is the most frequently utilized donor site, providing corticocancellous bone that integrates effectively with the recipient site, achieving success rates exceeding 80% in clinical studies [7,8]. For instance, Hudak et al. documented a 92.9% success rate with calvarial bone grafts over a 25-year follow-up, underscoring the reliability of autologous sources [9]. However, the procedure’s drawbacks—such as donor-site morbidity (e.g., chronic pain, infection, or pelvic instability)—have driven exploration into alternative materials [5,6,8,10,11,12,13]. These limitations include prolonged recovery times and, in rare cases, complications necessitating additional surgery.

To circumvent these challenges, alternative grafting options such as allografts, xenografts, and synthetic bone substitutes have gained attention. Allografts, sourced from human cadavers, eliminate donor-site morbidity and are widely available, yet they lack viable osteogenic cells and carry a minimal risk of disease transmission [5,14]. Xenografts, typically deproteinized bovine bone, serve as osteoconductive scaffolds but lack osteoinductive capacity and raise concerns about immunogenicity or zoonotic disease transmission [5,14,15,16,17]. Synthetic grafts, such as hydroxyapatite or tricalcium phosphate, offer biocompatibility and customizable properties, but their lack of osteogenic and osteoinductive potential limits their regenerative efficacy, as shown in Table 1 [5,14,16]. While these alternatives reduce surgical burden, autologous grafts maintain superiority due to their unmatched biological performance, though research continues to refine substitutes for broader clinical applications [5,18].

Recent innovations in regenerative dentistry have expanded the toolkit for AC repair. The incorporation of growth factors, such as bone morphogenetic protein-2 (BMP-2) and platelet-rich plasma (PRP), enhances graft performance by accelerating osteogenesis and reducing integration time—the duration required for the graft to fuse with the host bone [19,20]. Li et al. demonstrated that PRP supplementation in autologous iliac grafts increased bone density by 6 months post-surgery, with fewer complications than grafts alone [19]. Similarly, BMP-2 has been shown to boost bone volume by up to 30% in preclinical models, offering a potent adjunct to traditional methods [21]. Beyond growth factors, stem cell therapy and tissue engineering—utilizing mesenchymal stem cells (MSCs) and biocompatible scaffolds—are emerging as transformative approaches [21,22,23]. These techniques aim to regenerate bone with minimal reliance on donor tissue, potentially revolutionizing outcomes in complex cases.

The timing of intervention is pivotal to treatment success. Secondary alveolar bone grafting (SABG), performed during the mixed dentition phase (ages 6–11), is widely accepted as the optimal period, coinciding with the eruption of the permanent canine [6,24,25]. This timing maximizes support for dental development while minimizing interference with maxillary growth, as evidenced by Chang et al. and Brudnicki et al., who linked SABG to improved craniofacial morphology and reduced revision rates [26,27]. However, individual variability, such as the patient’s skeletal maturity and cleft severity, requires personalized timing adjustments to optimize outcomes.

This review synthesizes current evidence on bone grafting modalities for anterior lip/palate cleft in children, spotlighting the integration of regenerative innovations like growth factors and stem cells. By weighing the strengths and limitations of autologous, allogeneic, xenogeneic, and synthetic materials, it aims to equip clinicians with a nuanced understanding of effective strategies for restoring function and aesthetics. Emphasizing a tailored, multidisciplinary approach, this article highlights the evolving landscape of cleft care and the promise of regenerative therapies in achieving superior long-term results.

## 2. Materials and Methods

This review was conducted through a search of scientific and dental articles published in databases such as PubMed, Medline, EBSCO, ELSEVIER, Cochrane, and Google Scholar. To achieve the research objective and have a clear guide, an electronic search was performed with specific filters to locate the maximum number of articles using the keywords described in Table 2.

In addition to applying an advanced search in the databases used, the inclusion and exclusion criteria for selecting suitable articles for this study are described in Table 3.

Table 3 summarizes each included article by author, year, and journal of publication, the aim and the test type, the results and the conclusions.

**Figure 1:**
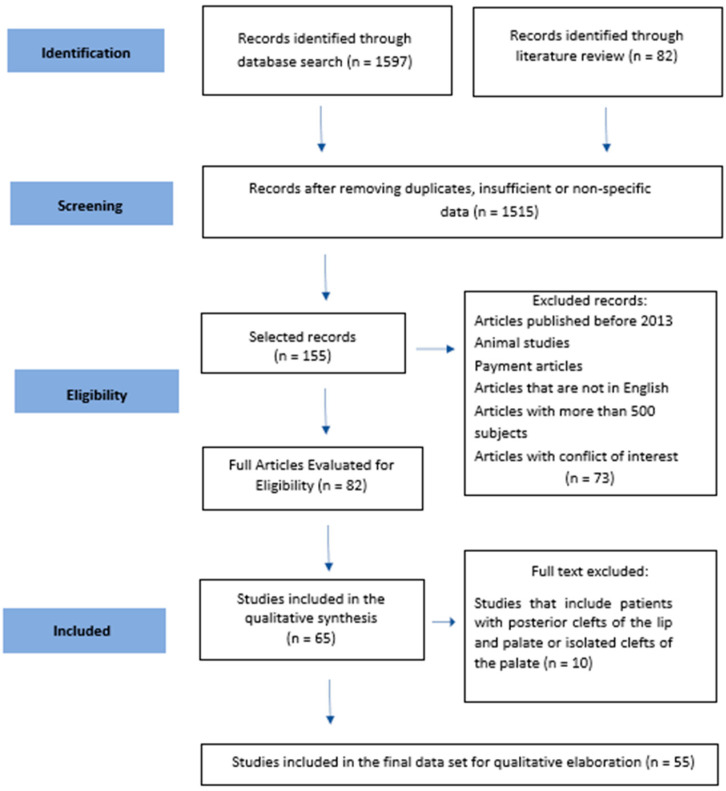
Search strategy flowchart.

Figure 1 describes the search strategy used in this study using a flow chart.

## 3. Results

During the literature review process, 53 articles were selected based on the objectives established at the beginning of the study and following the inclusion and exclusion criteria.

The process of identifying, selecting, and removing studies is summarized in an initial phase where 1597 studies were identified. Of this total, 82 articles were considered potentially eligible based on the title and general description.

In the second phase, 63 articles were selected based on the full-text analysis, and 10 of these articles were excluded due to the inclusion and exclusion criteria or because they were duplicates.

The relevant aspects of the remaining 53 articles, including authors, publication year, objectives, study type, description of results, and conclusions, are shown in Table 4.

## 4. Discussion

The treatment of anterior lip/palate cleft in children poses a complex challenge, requiring a deep understanding of regenerative treatment options, particularly bone grafting, to reconstruct the alveolar ridge and support craniofacial and dental development.

The management of patients with cleft lip and palate requires a multidisciplinary approach from birth to adulthood, involving multiple surgical and non-surgical interventions. One of the key procedures is the reconstruction of the alveolar cleft using bone grafts, as its presence generates functional, aesthetic, and psychological problems, affecting speech, swallowing, dental occlusion, facial development, and patient self-esteem [2].

According to authors Dissaux C et al. [5], McCrary H et al. [25], Matthews Zúñiga F et al. [12], among others [7,35,40], the main goal of regenerative treatment is to restore the continuity of the alveolar ridge, provide bone support for dental eruption and/or implant placement, close oronasal communications, improve nasal projection, and optimize outcomes of future corrective surgeries.

### 4.1. Family History and Risk Factors

Understanding the etiology of anterior lip/palate cleft is essential for tailoring regenerative treatments and informing preventive strategies, with family history and environmental risk factors playing significant roles.

Lekaviciene et al. emphasize a strong genetic component, reporting a markedly higher prevalence of cleft lip and palate among families with a history of the condition, suggesting that inherited factors contribute substantially to its occurrence [54]. This familial clustering aligns with broader genetic insights; Babai et al. note that advancements in genetics have identified multiple genes and syndromes linked to orofacial clefts, enhancing prenatal diagnosis and treatment planning [29].

However, genetics alone does not fully account for cleft etiology; environmental influences during pregnancy amplify the risk. Xuan et al. found a statistically significant association between maternal smoking and cleft lip with or without palate, with an odds ratio (OR) of 1.368 (95% CI: 1.259–1.486), indicating a 36.8% increased likelihood, alongside a dose-response effect in half the studies reviewed [32]. Similarly, Molina-Solana et al. identified maternal alcohol consumption as a risk factor, with an OR of 1.28 (95% CI not specified), alongside other factors like obesity (OR 1.26), stress (OR 1.41), and low zinc levels (OR 1.82), while folic acid intake reduced risk (OR 0.77) [33].

These findings, supported by Hammond et al., underscore the interplay between genetic predisposition and modifiable environmental exposures during embryogenesis [31]. For clinicians, this dual etiology necessitates thorough family history assessments and prenatal counseling to identify at-risk patients, potentially guiding the timing and type of bone grafting interventions. While regenerative treatments like autologous grafts remain the focus, addressing these risk factors could mitigate cleft severity and improve long-term outcomes, though further research into gene/environment interactions is needed [29,54].

### 4.2. Bone Grafting Materials: A Comparative Overview

Bone grafting is the cornerstone of alveolar cleft repair. Surgical techniques for alveolar reconstruction have evolved significantly over time, but the use of autologous bone grafts, especially from the iliac crest, remains the “gold standard” for many professionals. The main advantage of these grafts is their osteogenic, osteoinductive, and osteoconductive capacity [5,16], but their use implies an additional surgery and may generate morbidity at the donor site [5,6,8,10,11,12,13].

To avoid the morbidity associated with autologous grafts, alternatives such as allografts, xenografts, and synthetic grafts have been investigated. Allografts, such as demineralized bone, have osteoconductive and osteoinductive properties but lack live cells and may transmit diseases [5,16].

Xenografts, such as deproteinized bovine bone, are osteoconductive but not osteoinductive [5,15], and synthetic grafts, such as hydroxyapatite and tricalcium phosphate, provide an osteoconductive scaffold but lack osteogenic and osteoinductive properties [14,18,29,41].

Recently, the use of tissue engineering techniques and cell therapies to improve bone regeneration in alveolar cleft defects has been explored [18,21,22,23,42]. The combination of stem cells, growth factors, and biomaterials has shown promising results in preclinical and clinical studies [21,22,23,43,44,45].

The use of cells derived from bone marrow, adipose tissue, umbilical cord, and periosteum to promote bone formation has been reported [22,43,44,45]. Additionally, the use of platelet concentrates, such as platelet-rich plasma and platelet-rich fibrin, has been shown to improve the quality and quantity of grafted bone [19,20].

The choice of material depends on factors such as availability, donor site morbidity, surgeon preferences, and the optimal timing for performing the alveolar bone graft. Some studies suggest that grafting prior to orthodontic treatment may improve craniofacial morphological outcomes [26,27,46].

Nevertheless, it is classically proposed to perform the graft between 7 and 11 years of age, during the eruption of the permanent canine to optimize graft stability and reduce the need for additional procedures [6,24,47]. However, other authors suggest an earlier approach, performing the graft between 5 and 7 years or even in pre-surgical stages through primary and secondary gingivoperiosteoplasty [14,26,36].

### 4.3. Long-Term Stability of Grafts

Long-term stability is vital for maintaining functional and aesthetic results in alveolar cleft repair, yet data beyond five years are scarce for many materials.

Hudak et al. reported a 92.9% success rate with calvarial grafts over 25 years, with minimal resorption [9]. Wang et al. found implant survival rates of 80.7% to 96.3% in grafted sites after 1 to 5 years, suggesting good initial stability [3], and Kang noted that iliac crest grafts maintain volume, though resorption can range from 10% to 50% without proper stabilization [6].

Non-autologous materials lack comparable long-term evidence. Aly et al. reported promising bone formation with bovine xenografts at 6 months [15], and Al-Rawee et al. found effective reconstruction with synthetic Osteon III at 6 months [14], but neither study extended beyond one year. This highlights a critical research gap, especially for pediatric patients whose craniofacial growth continues, necessitating durable solutions.

### 4.4. Role of Growth Factors and Stem Cells

Emerging regenerative therapies, such as growth factors and stem cells, enhance bone graft outcomes by accelerating osteogenesis, improving integration, and potentially reducing the need for large autologous harvests.

BMP-2: Francisco et al. found no significant difference in bone volume between BMP-2 and iliac crest grafts after 6–12 months (*p* = 0.704), though BMP-2 shortened hospital stays [42]. Scalzone et al. noted increased bone formation at 6 months with BMP-2 (MD −14.410, *p* = 0.000), though benefits faded by 1 year [16].

PRP/PRF: Li et al. showed that PRP with autografts increased bone density at 6 months and reduced complications [19]. Shawky et al. (2016) found PRF improved bone formation (82.6% vs. 68.38%, *p* < 0.05), though density differences were not significant [20].

Khojasteh et al. reported that buccal fat pad-derived stem cells with iliac crest grafts achieved 82.5% bone formation vs. 70% in controls [45], and Mazzetti et al. observed that umbilical cord stem cells reduced inflammation and fibrosis, aiding recovery [44].

These biologics show promise, particularly in early healing, but their long-term benefits and standardization require further exploration via RCTs.

### 4.5. Impact of Timing and Age on Graft Outcomes

Patient age is also an important factor in planning, as it influences the selection of surgical technique, graft type, and bone regeneration potential. Timing significantly affects graft success, with secondary alveolar bone grafting (SABG) during mixed dentition (ages 6–11) considered optimal due to alignment with canine eruption and minimal impact on maxillary growth [6,24,25].

Primary ABG (0–6 years): Wang et al. reported a 72% success rate with primary gingivoperiosteoplasty, but larger residual defects suggest higher resorption [36].

As explained by Chang C et al. [26], in patients under 6 years old, the use of early secondary bone grafts with gingivoperiosteoplasty and pre-surgical orthopedics has gained popularity. This allows the utilization of the high regenerative potential at early ages and early guidance of alveolar ridge development. Nonetheless, spontaneous closure of small alveolar defects after hard palate repair has also been described in some studies, such as the study by Scheuermann M et al. [51].

In pediatric patients (6–11 years) or secondary ABG: Kang found 68–71% of grafts achieved normal bone height at 1 year [6], but Kaura et al. noted better outcomes in older children within this range of 7–12 years [24].

On the other hand, in adolescent and/or adult patients, for late Secondary ABG (>11 years), Green et al. observed that grafting at 10.1 years required more revisions than at 12.3 years (*p* < 0.001), indicating greater stability with age [47]. That means that late alveolar reconstruction may be necessary when not performed in childhood or if residual defects persist. In these cases, the goals are to improve aesthetics and enable prosthetic rehabilitation, generally with dental implants [3,4,52].

### 4.6. Comparison of Surgical Techniques: Iliac Crest vs. Calvarial Grafting

Choosing between iliac crest and calvarial grafting involves trade-offs. Iliac crest is described as having a success rate of around 96.7% with favorable outcomes [7]. It also shows 5–10% complications because of donor-site morbidity (pain, instability) [10,11]. In the end, the patient experience is notable for pain and recovery time [8]. On the other hand, calvarial grafts have a success rate of 92.9% over 25 years. Complications are around 1% morbidity with limited volume, and the patient experience involves less pain and better cosmesis [9].

Intraoral autologous grafts (ramus/chin) are preferred for their low morbidity, although extraoral grafts (iliac crest) remain a valid and well-studied option. The use of guided bone regeneration techniques with membranes and particulate materials has shown good results in localized defects [48,50].

### 4.7. Surgical Considerations

Other important aspects to consider in alveolar reconstruction are the type of incision, the use of barrier membranes, and graft stability. It has been reported that an incision limited to the keratinized gingiva improves the qualitative outcomes of the bone graft [48], and the use of barrier membranes such as absorbable collagen can improve graft stability and reduce future bone resorption [29,49,50], thus avoiding additional surgeries before implant placement. Therefore, proper stabilization of the graft with osteosynthesis plates and screws is essential to prevent displacement and improve outcomes [5,6,49].

### 4.8. Common Complications of Bone Grafting

It is important to mention that alveolar reconstruction with bone grafts is generally a safe procedure, with reported complication rates between 5% and 10% [25,40]. However, it is crucial to consider the associated risks and morbidities. Harvesting autologous grafts can generate various complications at the donor site, such as pain, paresthesia, hematoma, infection, scarring, and gait disturbances when obtained from the iliac crest, facial asymmetry in the case of the chin, or trismus and nerve injury when extracted from the mandibular ramus. These complications can be avoided using allogenic, xenogenic, or synthetic grafts [5,6,8,10,11,12,13].

Another possible complication is infection, which can occur at both the recipient and donor sites. Risk factors include poor oral hygiene, persistence of oronasal communication, and use of non-biocompatible materials. Treatment to eliminate infections involves antibiotics, drainage, and, in some cases, removal of the graft [32,40].

The most frequent complication is bone resorption following surgery, reported as partial or total in 10% to 50% of cases, being higher when extraoral autologous grafts are used [10,12,16]. Factors such as advanced age, smoking, poor graft stabilization, and lack of medullary bone graft are associated with this complication. The use of alveolar preservation and bone regeneration techniques can help reduce resorption [37,49].

Dehiscence and graft exposure is another complication that can occur due to tension closure of soft tissues, infection, or necrosis. Management requires the use of local flaps, soft tissue grafts, or covering materials such as membranes to prevent graft loss [49,50].

Finally, despite adequate alveolar reconstruction, teeth adjacent to the cleft, such as the lateral incisor and canine, may become impacted or exhibit ectopic eruption. In these cases, orthodontic traction or eventual tooth extraction is required [30,36].

To avoid various complications or improve future outcomes, regenerative therapies, including the use of olecranon grafts [11], mineralized plasma matrices [53], and resorbable collagen membranes [37,49] have shown promising results, but more clinical trials are needed to establish their long-term efficacy and safety.

### 4.9. Future Directions

The rehabilitation of patients with alveolar cleft through dental implants after bone grafting has been extensively studied in the literature. Wermker et al. [4] and Vuletić et al. [35] conducted studies that evaluated the survival and success of dental implants in patients with grafted alveolar cleft, obtaining similar results. Vuletić et al. observed a survival rate of 93.3% and a success rate of 86.7% after a mean follow-up of 5 years, while Wermker et al. reported a weighted average survival rate of 91.2% and a success rate of 82%. These findings are supported by the systematic review of Wang et al. [3], which included 14 studies and found a weighted average survival rate of 95.5% and a weighted average success rate of 91.5% for implants placed in patients with alveolar cleft.

Van Nhan et al. [34] and Khojasteh et al. [45] presented new alveolar bone grafting techniques to improve implant placement outcomes in cleft patients. Van Nhan et al. used a combination of iliac crest block bone graft and maxillary tuberosity particulate bone graft, achieving a 100% implant survival rate after a mean follow-up of 30 months. On the other hand, Khojasteh et al. employed a combined technique of autogenous bone graft, flap-derived cells of Bichat, and a cortical bone plate from the mandibular ramus, also achieving a 100% implant survival rate and an average vertical bone gain of 9.4 mm after a 12-month follow-up.

Pucciarelli et al. [52] presented a clinical report and guidelines for implant placement in patients with cleft lip and palate, highlighting the importance of a thorough pre-operative evaluation, adequate healing time after bone grafting, and the use of guided bone regeneration techniques when necessary. These recommendations are consistent with the approaches used in the studies by Van Nhan et al. and Khojasteh et al., which achieved excellent results in implant therapy in patients with alveolar cleft.

### 4.10. Future Perspectives on Bone Grafting and 3D Printing

The future of bone grafting for treating ALPC holds immense promise through the synergy of regenerative medicine and 3D printing technologies. Patient-specific 3D-printed scaffolds, crafted from biocompatible materials such as hydroxyapatite (a mineral naturally found in bone or polycaprolactone, a biodegradable polymer, enable unprecedented precision in graft design). These materials can be tailored to replicate the complex geometry of the alveolar defect, ensuring a perfect fit that enhances both functionality and aesthetics [41]. This level of customization significantly reduces donor-site morbidity, a longstanding drawback of traditional autologous grafts harvested from sites like the iliac crest or calvarial bone, which often result in pain, infection risk, or limited tissue availability [5,6,9,11].

Furthermore, these scaffolds can be enhanced with bioactive molecules like bone morphogenetic proteins (BMPs), which trigger osteoblast differentiation to form new bone, and vascular endothelial growth factors (VEGFs), which stimulate blood vessel formation to support tissue regeneration [21,22,23]. The incorporation of stem cells, such as mesenchymal stem cells (MSCs) derived from adipose tissue or bone marrow, adds another layer of potential by providing a renewable source of bone-forming cells. Preclinical studies, such as the work by Martín del Campo et al., have demonstrated this potential, achieving significant bone regeneration in animal models using 3D-printed scaffolds seeded with stem cells [23].

As researchers committed to improving ALPC treatment, we view the convergence of 3D printing and regenerative medicine as an innovative shift that could redefine patient care. The ability to design customized, bioactive grafts that restore both form and function while minimizing the surgical burden (particularly for young patients facing multiple interventions) fills us with excitement and hope. Imagine a future where a child with ALPC receives a single, tailored graft that grows with them, reducing the need for repeated surgeries and improving their quality of life. However, we are equally aware of the barriers that lie ahead. Ensuring the long-term safety and efficacy of these technologies, especially in pediatric patients whose bones are still developing, requires extensive clinical validation through large-scale, longitudinal trials [18,41]. Beyond the science, practical challenges stand out as major concerns, including the high cost of 3D printing equipment and materials, along with regulatory complexities surrounding new biomaterials, which could limit accessibility, particularly in under-resourced healthcare systems.

Despite these obstacles, our optimism endures. We believe that with sustained interdisciplinary collaboration (bringing together engineers, clinicians, and representatives) and ongoing technological refinement, 3D-printed regenerative grafts will soon become a cornerstone of ALPC treatment. This future promises a paradigm where cleft repair is not only less invasive and more predictable but also uniquely adapted to each child’s needs, paving the way for more equitable and effective care worldwide.

## 5. Conclusions

In conclusion, alveolar reconstruction in patients with alveolar clefts remains a complex challenge that requires a multidisciplinary approach. Although autologous bone grafting remains the standard, regenerative therapies and biomaterials are emerging as promising alternatives. The choice of the optimal timing for grafting and the surgical technique should be tailored to each patient, but it is described that during the eruption of the canine tooth is the timing that gains the most advantages. Advances in 3D technology and implant rehabilitation have improved long-term outcomes, and the studies analyzed show that dental implant placement in grafted alveolar cleft patients is a predictable and successful treatment option, with implant survival and success rates exceeding 90% in most cases. The use of advanced bone grafting techniques, such as the combination of block and particulate grafts or cellular therapy, can further improve outcomes. Through pre-operative planning, adequate healing time, and the use of guided bone regeneration techniques when necessary are key factors for the success of implant therapy in these patients. Nevertheless, more research is needed to refine surgical techniques, explore new regenerative therapies, and establish evidence-based protocols to optimize functional and aesthetic outcomes in patients with alveolar clefts.

## Figures and Tables

**Table 1:** Structured comparison of bone graft materials.

Graft Type	Osteogenic Potential	Integration Time	Clinical Outcomes	Advantages	Disadvantages
Autografts (e.g., iliac crest, calvarial)	High (contains live cells)	3–6 months	High success rates (e.g., 92.9% for calvarial grafts)	Osteogenic, osteoinductive, osteoconductive	Donor-site morbidity (pain, pelvic instability)
Allografts (e.g., demineralized bone)	None (no live cells)	6–12 months	Variable, less predictable than autografts	No donor-site morbidity, widely available	Risk of disease transmission, lower osteoinduction
Xenografts (e.g., bovine bone)	None (osteoconductive only)	6–12 months	Good bone formation	Biocompatible, osteoconductive scaffold	Risk of cross-species disease, slow resorption
Synthetic grafts (e.g., hydroxyapatite tricalcium phosphate)	None (osteoconductive only)	6–12 months	Effective scaffold	No morbidity, customizable	No osteoinduction, variable long-term stability

**Table 2:** Keywords used for research.

Keywords	Number of Articles Found	Number of Articles Used
“Cleft lip” AND “Cleft palate”	1271	8
“Cleft palate” AND “Embryology”	74	3
“Cleft palate” AND “Aetiology” AND “Epidemiology”	107	4
“Cleft palate” AND “Bone graft”	36	12
“Cleft palate” AND “Alveolar bone grafting”	62	18
“Secondary alveolar bone grafting”	47	8
TOTAL	1597	53

**Table 3:** Inclusion and exclusion criteria used for research.

	Inclusion Criteria	Exclusion Criteria
**Publication Date**	2013–2023	Articles published before 2013
**Language**	English Spanish	Any other language
**Type of Study**	Experimental studies, Literature reviews, Systematic reviews, In vivo/In vitro studies, Clinical cases and studies	Animal studies, Theses, Books, and Payment articles (not available online)
**Population**	Patients with cleft lip and palate without associated syndromes	Patients with cleft lip and palate with associated syndromes
**Number of Participants**	No minimum required	Maximum studies with groups of 700 children
**Geographic Location of the Study**	Not specified	-
**Intervention**	Use of bone grafts or substitutes for alveolar reconstruction in cleft lip and palate	Studies that include patients with posterior clefts of the lip and palate or isolated clefts of the palate
**Conflict of Interest**	Articles without conflict of interest	Articles with any conflict of interest to avoid biased results

**Table 4:** Summary of each article included in the review, dividing by author, year and journal of publication, study type, aim, test type, results, and conclusions.

Author	Year	Journal	Study Type	Aim	Test Type	Results	Conclusions
Marginean C. et al. [28]	2018	*Medical Ultrasonography*	Clinical Case	To present the particularities related to the prenatal diagnosis of orofacial clefts (gestational age at the time of diagnosis, type, and the presence of other associations) in a series of 11 cases.	In Vivo	Eleven fetuses (0.13%) were diagnosed by 2D and 3D ultrasound of CL (4 cases) or CLP (7 cases). The lowest gestational age at the time of diagnosis was 14 weeks, while the highest was 35. Of the 7 cases diagnosed with CLP, 4 also had other associated anomalies. All 4 CL cases had identifiable associated anomalies. In 3 cases of CLP, pregnancy termination occurred.	Prenatal diagnosis of orofacial defects is continually improving. CLP can be diagnosed even at the end of the first trimester of pregnancy. LC is usually diagnosed during a routine 2nd trimester ultrasound and is usually an isolated abnormality.
Babai A. et al. [29]	2023	*Genes*	Systematic Review	To summarize the multiple genetic etiologies of orofacial clefts, examine several common syndromes, and address strategies for the management of these clefts.	-	Does not present results	Progress in genetics has driven the discovery of genes and syndromes associated with orofacial clefts, improving prenatal diagnosis, early treatment, and quality of life, although more research is required on the gene/environment interaction for prevention.
Li T. et al. [19]	2023	*International Journal of Oral & Maxillofacial Surgery*	Systematic review and meta-analysis	To review the existing evidence from randomized clinical trials (RCTs) on the effect of autogenous bone grafts combined with a platelet-rich concentrate on alveolar clefts.	-	Platelet-rich plasma (PRP) increased bone density in autologous alveolar cleft grafts vs. autologous bone graft alone at 6 months. Fewer complications occurred with autologous bone graft plus platelet-rich plasma (PRP) vs. autologous graft alone.	This study did not show benefits from the use of platelet-rich plasma together with autologous bone graft in alveolar fissures with respect to bone volume, bone density, or complications.
Dissaux C. et al. [5]	2022	*The Cleft Palate-Craniofacial Journal*	Systematic Review	To examine the various alternatives for bone filling and to compare their effectiveness with respect to the results of the implant of cancellous tissue extracted from the iliac crest (ICBG).	-	Bone grafts are classified as autologous, materials complementary to iliac crest cancellous bone graft (ICBG), and bone substitutes. No autologous agent showed greater efficacy than ICBG, although this is inconsistent and causes morbidity. Adding platelet concentrates to ICBG has only shown advantages in 3 investigations. 3D evaluations of scaffolds are scarce. One study showed improvement when adding a cellular dermal matrix, and cellular therapies, despite being promising, remain limited.	ICBG continues to be the gold standard of reference in alveolar bone grafts, although some alternative materials have achieved similar results. Its integration process and the effect of mechanical forces are poorly understood, so there is a need to study and understand the mechanics of mechanotransduction in the osseointegration of grafts.
Jabbari F. et al. [30]	2016	*Upsala Journal of Medical Sciences*	Cohort study	To investigate the possible relationship between the size of the cleft at birth and the long-term decrease in alveolar bone height, as well as the presence of dental anomalies in patients with cleft lip or palate.	Comparative In vivo Longitudinal	**Height of the alveolar bone:** There was no significant correlation between initial cleft width and alveolar bone height at either 1 or 10 years of follow-up. **Teeth condition:** There was no correlation between enamel hypoplasia and posterior cleft width. **Canine inclination:** There was no correlation between the width of the fissure and the inclination of the canine. **ICS Rotation and Tilt:** Central incisor rotation was positively correlated with the relative width of the anterior cleft. **Lateral incisor:** There was lateral hypodontia in 20 patients. The width of the posterior fissure was positively correlated with the presence of laterals, so wider fissures were associated with the presence of laterals.	The initial fissure measurement has no apparent impact on the results of posterior alveolar bone grafting; however, there is a positive correlation between the severity of central incisor rotation and dental enamel hypoplasia. Therefore, the initial fissure size could serve as an early indication to identify the possible need for more intensive orthodontic treatment in the context of secondary alveolar bone grafting.
Wang F. et al. [3]	2014	*International Journal of Oral & Maxillofacial Implants*	Systematic Review	To analyze the evidence available in the literature to establish the clinical results of implant therapy combined with bone grafting in patients affected by alveolar cleft.	-	The survival rate of implants varied between 80.7% and 96.3%, with a follow-up of 1 to 5 years. The most frequent complications were marginal bone loss, peri-implantitis, and lack of osseointegration. Implants placed in the cleft area had a lower survival rate compared to implants in unaffected areas. The use of bone graft and the surgeon’s experience positively influenced the results.	The evidence on dental implants in patients with alveolar cleft is varied. In the short term (<5 years), combining implants with bone grafts seems predictable. More long-term data (>5 years) on success rates and peri-implant health are needed to evaluate its effectiveness over time.
Wermker K. et al. [4]	2014	*International Journal of Oral & Maxillofacial Implants*	Systematic Review	To evaluate the long-term survival of dental implants placed in patients with cleft lip and palate, as well as to determine the most appropriate time for their insertion after performing an alveolar bone graft in the area.	-	The methodological quality of the studies varied, with evidence levels 3b and 4 predominating, reflecting a moderate to low quality. The five-year implant survival rate ranged from 80% to 96%, with an average of 88.6%. Generally, implant placement is recommended after the patient’s growth and usually between 4 and 6 months after alveolar bone grafting.	Dental implants in patients with cleft lip and palate show high success rates and facilitate adequate oral rehabilitation. However, the available evidence is limited and of low quality due to the paucity of prospective clinical studies on dental implants in this specific population.
Hammond NL et al. [31]	2022	*Oral Diseases*	Literature Review	To carry out an updated review of the existing literature on how the lip and palate develop during embryonic life.	-	Does not present results.	Cleft lips and palates are common congenital facial malformations that result from a failure to fuse embryonic facial processes, causing significant feeding, speech, and health problems. It is crucial to study facial embryogenesis to understand its molecular pathogenesis and develop better treatments.
Xuan Z. et al. [32]	2016	*Oral Surgery, Oral Medicine, Oral Pathology and Oral Radiology*	Meta-analysis	To evaluate the association between maternal active smoking and the risk of oral clefts (birth defects) in offspring.	Case-control and cohort studies	A modest but statistically significant association was found between smoking during pregnancy and the risk of having a child with cleft lip with or without cleft palate (CL ± P) and cleft palate alone (CP). Odds Ratio (OR) for CL ± P: 1.368 (95% Confidence Interval [CI]: 1.259–1.486) OR for CP: 1.241 (95% CI: 1.117–1.378). Half of the studies showed a positive dose-response effect for each subgroup.	Despite the variability in studies, the interaction between genetics and environment possibly best explains the effect of smoking on pregnancy. Evidence suggests that smoking during pregnancy negatively affects childbirth, underscoring the importance of not smoking during this period.
Salari N. et al. [1]	2022	*Journal of Stomatology, Oral and Maxillofacial Surgery*	Systematic Review and Meta-analysis	To determine the global prevalence of cleft palate, cleft lip, and cleft lip and palate.	Statistics	The prevalence of cleft palate based on the studies reviewed per 1000 live births was obtained as 0.33 (95% CI: 0.28–0.38). The prevalence of cleft lip from the studies reviewed was 0.3 per 1000 live births (95% CI: 0.26–0.34). The prevalence of cleft lip and palate in the studies reviewed per 1000 live births was 0.45 (95% CI: 0.38–0.52).	Due to the high prevalence of oral clefts, those responsible for the health system must take preventive measures to reduce the number of patients, as well as diagnostic and therapeutic tests to reduce the effects of this disorder in children.
Molina-Solana R. et al. [33]	2013	*International Journal of Oral and Maxillofacial Surgery*	Meta-analysis	To explore how the incidence of cleft lip and/or palate may be influenced by environmental factors such as tobacco and alcohol consumption, folic acid intake, obesity, stress, low levels of zinc in blood, and fever during pregnancy.	Case-control, cohort, and cross-sectional studies	The most associated maternal factors were tobacco (OR 1.48), alcohol (OR 1.28), folic acid intake (OR 0.77), obesity (OR 1.26), stress (OR 1.41), low levels of zinc in the blood (OR 1.82), and fever during pregnancy (OR 1.30). Folic acid intake reduced the risk of cleft lip and palate in offspring (OR 0.77).	The study highlights the importance of controlling environmental factors during pregnancy to reduce the prevalence of cleft lip and palate.
Van Nhan V. et al. [34]	2018	*The Cleft Palate-Craniofacial Journal*	Non-randomized prospective clinical trial	To evaluate a new bone graft technique with two cortico-cancellous iliac crest blocks for the placement of dental implants in patients with cleft lip and palate.	In vivo clinical test	Of the 32 patients, 90.6% showed 75% to 100% bone filling according to the Enemark scale. The mean height and width of the graft were 11.4 ± 2.4 mm and 6.1 ± 1.0 mm, respectively. A total of 90.6% of patients showed sufficient bone for implant placement. Minor complications such as flap dehiscence were observed in 9.4% of patients without associated infections.	The suggested technique, which uses two corticocancellous blocks of the iliac crest, is effective and safe for the reconstruction of the alveolar cleft, thus facilitating the placement of dental implants in patients with cleft lip and palate.
Cho-Lee G. et al. [7]	2013	*Annals of Maxillofacial Surgery*	Retrospective study	To evaluate the protocol, surgical technique, and outcomes of secondary alveolar bone grafting in patients with alveolar cleft at a single institution.	Descriptive analysis	One hundred nine procedures were performed in 90 patients with alveolar cleft. The average age was 14.2 years, and in all cases, an iliac crest graft was used. The success parameters were favorable in 87 patients; there were only 3 cases of graft loss. In another 3 cases, previously used allogeneic materials became infected and were replaced with an iliac crest graft.	The use of autogenous iliac crest bone graft for secondary repair of alveolar fissure achieves multiple objectives, providing optimal support and allowing stabilization of the maxillary segments, among others. The use of cortico-cancellous block grafts combined with bone chips is recommended to optimize results.
Vuletić M et al. [35]	2014	*Acta Stomatologica Croatica*	Literature Review	To explain the morphology of defects, the historical perspective, surgical techniques, and the possibilities of implantological and prosthodontic rehabilitation.	-	Does not present results.	Bone grafts are crucial for the management of alveolar defects, allowing for better aesthetics and function. Autogenous iliac crest bone remains the gold standard, although it is not ideal in all cases. Alternatives such as BMPs offer new possibilities, although more studies are needed to validate their long-term efficacy and safety. Implant/prosthetic rehabilitation is feasible, although success varies and may require additional orthodontic management.
Yates D. et al. [2]	2020	*Oral and Maxillofacial Surgery Clinics of North America*	Literature Review	To provide an overview of the timeline and progression of interventions involved in the comprehensive care of patients with cleft lip and/or palate from birth to adulthood.	-	Pre-surgical children’s orthopedic treatment helps improve nasal and lip anatomy before surgery. Surgical repair of cleft lip usually occurs between 3 and 6 months to improve feeding and future speech. Primary palatal repair occurs between 10 and 18 months until speech development requires closure. Velopharyngeal insufficiency occurs in 5–40% of cases, causing nasal leakage and hypernasality. Maxillary expansion is performed about 6 months before grafting to allow access to the site. The timing of grafting depends on the eruption of the canines and the proximity of the fissure to the developing roots. Maxillary bone grafting should be performed before teeth erupt at the crack site, between 5 and 11 years of age. Autogenous iliac crest graft is the standard, recently challenged by BMP with allograft. In bilateral clefts, it may be necessary to reposition the premaxilla at the time of bone grafting. Due to the blood supply, only one side is grafted at a time.	It highlights the importance of a multidisciplinary team approach, starting with pre-surgical orthopedic treatment in infants, followed by cheiloplasty and primary palatoplasty within the first year. Subsequently, procedures such as alveolar bone grafting, rhinoplasty, and orthognathic surgery are performed at the appropriate time during childhood and adolescence. Adequate timing and coordination between specialties are crucial in the management of these complex anomalies.
Kang NH [6]	2017	*Archives of Plastic Surgery*	Literature Review	To review current methods for the treatment of alveolar fissures.	-	SBG between 6 and 11 years has better survival and fewer negative effects on facial growth than primary or late grafts. Autologous cancellous bone from the iliac crest has good results as a biomaterial. Bone substitutes present different degrees of resorption, with bovine HA having the lowest density compared to autograft. Recombinant bone morphogenetic proteins show potential, but their use is not yet widespread. The main complications are graft exposure, excessive resorption, and failure requiring reintervention. Secondary graft survival at 1 year is 68–71% type I (normal bone height) and 15% type II (75% normal height).	The approach to treatment involves the use of the patient’s own cancellous bone grafts, taken from the iliac crest, in children aged 6 to 11 years during the mixed dentition phase. An alternative to autologous bone grafts combines demineralized allogeneic bone, which promotes new bone formation, with mineralized bone, which directs bone growth, resorbs slowly, and maintains its volume well. Additionally, technological innovations are being recognized for decreasing complications at the bone extraction site and are particularly beneficial when there is a shortage of bone available or in more complex clinical situations.
Chang C. et al. [26]	2016	*Scientific Reports*	Randomized, prospective, single-blind clinical study	To assess whether pre-operative orthodontic treatment affects the results of SABG in patients with unilateral cleft lip and palate.	Comparative	**Orthodontic Treatment:** —Volume of the alveolar bone defect before 1.41 ± 0.26 cm3 vs. after 1.10 ± 0.08 cm^3^. —Inclination of the ICS on the cracked side before 30.51 ± 6.33° vs. after 10.67 ± 0.50°. —Rotation of the ICS adjacent to the fissure before 41.43 ± 4.30° vs. after 10.54 ± 1.98°. **Measurements before SABG:** —The volume of the alveolar bone defect before surgery for the experimental group was similar to that of the control group. —The inclination and rotation of the ICS on the fissure side in the experimental group was less than in the control group. **Measurements six 6 after SABG surgery:** —The alveolar bone graft volume of the experimental group was greater than that of the control group. —The residual alveolar bone defect of the experimental group was lower than that of the control group. —The inclination and rotation of the ICS on the fissure side in the experimental group was significantly less than in the control group.	Orthodontic treatment combined with SABG results in superior bone volume compared to conventional SABG alone. It also improves the alignment and position of the teeth and can contribute to a higher success rate in alveolar bone grafting.
Dasari MR. et al. [8]	2018	*Contemporary Clinical Dentistry*	Longitudinal clinical study	To evaluate the success in repairing alveolar fissures with iliac bone grafts in addition to restoring the function and shape of both arches with an adequate occlusal relationship and tooth eruption in the fissure area.	Observational and longitudinal	Follow-up: two patients for 18 months, two patients for 12 months, and one patient for 6 months. Two patients showed more than 3/4 bone height after evaluation, another two had less than 3/4 height, and only one patient had complete graft failure. Two patients noted pain, slight bleeding, and slight swelling for approx. 24 h after the operation, but there were no post-operative complaints from the other two patients. Two patients were discharged the day after surgery, and two others two days later.	Secondary alveolar bone graft improves maxillary stability, supports dental eruption, and improves facial aesthetics, being optimal between 9 and 11 years of age. Autogenous iliac bone grafts provide abundant cancellous bone and reduce surgical time but may cause donor site morbidity. These drawbacks can be reduced with bone morphogenetic proteins and other combined materials.
Kaura AS et al. [24]	2018	*Journal of Craniofacial Surgery*	Systematic Review	To determine the optimal moment to perform a bone graft in the alveolar defect of patients with alveolar and palatal clefts.	-	Most of the studies reviewed suggest performing the bone graft in the alveolar gap before the permanent canines erupt, while others propose doing it in an age range from 7 to 12 years. The most common post-operative problems were wound dehiscence and infections in the treated area. Many studies applied additional treatments around surgery, especially orthodontic adjustments to prepare or improve the jaw. There was no direct relationship between patient age and graft success, but there was a slight trend toward better results with older patients.	Successful results of bone grafts in maxillary alveolar fissures are common, although the ideal time to perform them is not clear. Chronological age has not been shown to be a decisive factor. On the other hand, surgical preparation, technique, and interdisciplinary cooperation seem to be more decisive for success. Previous studies may confuse success with early graft age due to these factors. A randomized controlled study would help clarify the best time for grafting.
Wang Y. et al. [36]	2016	*Plastic and Reconstructive Surgery*	Prospective study	To compare the effects of primary gingivoperiostioplasty and secondary alveolar bone grafting on four outcomes: clinical success, Bergland scale score, residual cleft defect, and periodontal bone support of teeth adjacent to the cleft.	Comparison Clinical, level of Evidence III	Patients with primary gingivoperiostioplasty required more additional bone grafts compared to those who underwent secondary alveolar bone grafting. The residual cleft defect was greater in patients who underwent primary gingivoperiostioplasty compared with secondary alveolar bone graft. Residual coronal and apical palatal defects were more common in patients with primary gingivoperiostioplasty.	Secondary alveolar bone grafting is more effective than primary gingivoperiostioplasty in the treatment of unilateral cleft lip and palate, with a 72% success rate for the latter. Primary gingivoperiostioplasty tends to leave a larger residual defect in the alveolar cleft, especially in the apical part of the palate. However, both procedures provide comparable periodontal bone support for the teeth adjacent to the cleft.
Sleman N. et al. [10]	2023	*Annals of Medicine & Surgery*	Case Report	Demonstrate a novel technique for the repair of alveolar fissures, incorporating the benefits of previous studies in a single clinical case.	In vivo	Using a block graft technique evidenced by CBCT, the disappearance of the oronasal fistula and the continuity of the alveolar bone of the maxilla were achieved, which will make possible the restoration of the lost lateral incisor in the future through the placement of an implant.	Alveolar bone rehabilitation in patients with cleft lip and palate is essential to improve quality of life. This study proposes an effective surgical solution to achieve long-lasting, satisfactory results.
Hudak KA et. al. [9]	2014	*Plastic and Reconstructive Surgery*	Retrospective study	To evaluate the complications and outcomes of all patients undergoing alveolar skull bone grafts over a 25-year period, performed by a single surgeon, and provide an objective analysis of success.	Case series and cohort studies	In 308 patients with an average age of 11.5 years. Complications affected the graft extraction in 3.5%, the donor area in 1%, and the recipient area in 17.2%. A new graft was necessary in 7.1%, with a clinical success rate of 92.9%. The average volume of the alveolar defect was 1.19 mL before the operation and 0.19 mL after, achieving 85% filling of the defect according to radiological analysis.	Cranial bone graft is an effective alternative to iliac crest graft to treat alveolar cleft. This procedure uses membranous bone from a nearby donor area, which reduces morbidity and post-operative pain and leaves a barely visible scar. Given its advantages, cranial bone grafting should be considered more frequently as a viable treatment option for patients with alveolar cleft.
Singkhorn T. et al. [37]	2023	*International Journal of Oral & Maxillofacial Surgery*	Randomized clinical study	To investigate how a resorbable collagen membrane in secondary alveolar bone graft (SABG) treatment affects the quality and volume of the grafted bone in the cleft socket.	In vivo	The standard group without membrane (St) showed a significantly greater volume reduction than the group with membrane (Mb). The decrease in % density in the St group was notably greater than that in the Mb group after 1 and 3 months, but at 6 months, the differences were not significant. In the St group, bone graft quality was classified as good in 6 of 10 patients. In the Mb group, in 9 of the 10 patients.	This study confirmed that the use of a resorbable collagen membrane and the guided bone regeneration technique in the alveolar graft has advantages such as the preservation of bone volume, maintenance of contour, bone formation and maturation, in addition to facilitating dental eruption.
Aly LAA et al. [15]	2016	*Annals of Maxillofacial Surgery*	Clinical evaluative study	To clinically and radiographically evaluate the effectiveness of the 1:1 mixture of autogenous bone and deproteinized bovine bone, Bio-Oss, together with a resorbable collagen membrane for secondary closure.	In vivo	Excellent bone formation was observed at the treated sites, with an average increase of 5.45 mm (range 2–9 mm, standard deviation 1.93 mm).	Treatment of vertically deficient alveolar ridges with guided bone regeneration using a mixture of autogenous bone and DBBM with a resorbable collagen membrane is considered successful using this technique in an outpatient setting.
Al-Rawee RY et al. [14]	2023	*Archives of Plastic Surgery*	Prospective study	To examine the success rate of alveolar cleft grafting using bone substitutes such as Osteon III (Genoss) of different volumes and its relationship with time after surgery.	In vivo	A total of 55 participants between 6 and 13 years old (average: 9.2545 years); 25 men and 30 women. **Crack degree classification:** —Small (9 cases/16.3%); —Medium (20 cases/36.3%); —Large (26 cases/47.2%). Vol. 1 (baseline) = 18.1091 mm^3^. Vol. 2 (3 months) = 0.5109 mm^3^. Vol. 3 (6 months) = 22.5455 mm^3^. p-Highly significant value (0.005) paired sample *t*-test result (V1/V2). p-Highly significant value (0.001) paired sample *t*-test result (V2/V3).	A synthetic bone substitute, such as Osteon III, can be used for effective and uninterrupted reconstruction of the alveolar cleft.
Scalzone A. et al. [16]	2019	*Progress in Orthodontics*	Systematic Review and Meta-analysis	To conduct a review on the use of secondary alveolar bone graft (autologous bone and rh-BMP2 graft) to improve bone volume and height in patients with cleft lip and palate.	Statistics	Autologous bone graft showed statistically significant increased bone formation after a 6-month follow-up (MD −14.410, 95% CI −22.392 to −6.428, *p* = 0.000). No significant differences were observed after a 1-year follow-up (MD 6.227; 95% CI: −15.967 to 28.422; *p* = 0.582). No significant differences in bone height were observed after a follow-up of 6 months (MD—18.737, 95% CI—43.560 to 6.087, *p* = 0.139) and 1 year (MD −4.401, 95% CI −30.636 to 21.834; *p* = 0.742). Patients undergoing rh-BMP2 grafting had a significant reduction in hospital stay.	Autologous bone grafts and recombinant human bone morphogenetic protein demonstrated comparable effectiveness for maxillary alveolar reconstruction in patients with unilateral cleft lip and palate, with a shorter hospital stay for the protein graft, although with a high level of uncertainty.
Amiri MA et al. [22]	2022	*Journal of the Korean Association of Oral and Maxillofacial Surgeons*	Literature Review	To review the effectiveness of different sources of stem cells in bone regeneration of patients with cleft palate.	-	Does not present results.	The use of different types of stem cells, based on their accessibility and effectiveness in bone regeneration, is a promising method in bone regeneration of the cleft palate. Stem cells, despite the long procedures necessary for their cultivation and preparation, are a suitable alternative to conventional bone graft techniques.
Oliver JD et al. [21]	2021	*Tissue Engineering*	Literature Review	To review new molecular and cellular approaches in tissue engineering applied to cleft palate regeneration.	-	**Natural polymer scaffolds:** —Protein scaffolds: no specific clinical data are available for fissure repair. —Demineralized bone matrix: experiences in humans mean that the use and effectiveness remain controversial. —Polysaccharide scaffolds: limitation of their usefulness in tissue engineering. —Chitosan: has not yet been clinically evaluated. —Poly(hydroxyalkanoate)-based scaffolds: have not been described for the in vivo repair of fissures, but they have demonstrated their effectiveness in other bone regeneration applications. **Synthetic biomaterials for scaffolds:** —Bioactive ceramics: more studies in humans are needed. **Applications of growth factors:** —Bone morphogenetic proteins; —Growth factors derived from platelets; —Vascular endothelial growth factors; —Fibroblast growth factors; —Insulin-like growth factors; —Platelet-rich plasma and PRF; —Transforming growth factor-b. **Stem cell therapy:** —Bone marrow stem cells; —Stem cells from adipose tissue; —Dental stem cells.	There is great potential in the use of biomaterials and cell and gene therapies for tissue engineering applied to cleft palate. Biomaterials such as collagen, hyaluronic acid, and nanofibers are promising options as scaffolds to regenerate palatal tissues. Gene therapy is effective in modulating the expression of genes involved in tissue regeneration. Mesenchymal stem cells promote cell differentiation and regeneration. 3D printing allows customized scaffolds to be manufactured to repair specific defects. More research is required to optimize dosing and long-term effects and translate these new therapies into clinical applications.
Brauner E. et al. [17]	2018	*Clinical Therapeutics*	Retrospective study	To assess the aesthetic perception that patients had of themselves when comparing the placement of the implants with a third previous bone graft vs. only with a secondary graft.	Comparative analysis	In 14 patients: —Before any prosthetic rehabilitation, it was 4.6 (M = 5.8, F = 4). —After pre-surgical rehabilitation, the average score was 6.7 (M = 7.2, F = 6.4). —After rehabilitation with implants, it was 9 (M = 9.2, F = 9). A significant difference was calculated between patients who underwent tertiary grafting (score 9.5) and those who did not (score 8) (*p* < 0.01).	The findings indicate that while a secondary bone graft may be sufficient in some selected cases, ideal implant placement and better soft tissue adaptation are achieved with an additional tertiary graft, allowing for better aesthetic results and patient satisfaction.
Quintero González C. et al. [11]	2019	*Cirugía Plástica Ibero-Latinoamericana*	Descriptive and cross-sectional study	To describe and evaluate the results of the olecranon cancellous bone graft for the treatment of nasoalveolar fissure for more than 10 years.	Descriptive analysis	Surgical time ranged from 1 to 1.5 h. Return to daily activities ranged from 2 to 4 days post-operatively. Post-operative pain in the donor area was present in 5 patients (grade III-IV on a pain scale according to facies) and only in the first and second stages of grafting. At the time of bone grafting, 1 was 4 years old, 2 were 5 years old, 1 was 6 years old, and another was 8 years old.	The study describes the use of the olecranon region as a source of cancellous bone graft. Explains the modification of the technique carried out to obtain only cancellous graft, preserving the bone cortex to cover the bone window in the olecranon. According to the data, this favors low post-operative pain, reduction of hospital stay and complications, allowing rapid recovery. For this reason, they recommend the inclusion of this donor area as a resource for alveolar bone grafts.
Matthews Zúniga F. et al. [12]	2015	*Revista Médica Electrónica*	Literature Review	To describe the characteristics of the various alveolar graft techniques that are still the subject of controversy.	-	Does not present results.	Surgery should be performed during the period of mixed dentition before the eruption of the canine. Autologous bone constitutes the gold standard for this type of intervention, as does the iliac crest, the most promoted donor site. The cortico-medullary graft + the complementary use of rhBMP-2 has shown superiority to the trabeculated or cancellous graft.
Janjua OS et al. [38]	2022	*International Journal of Environmental Research and Public Health*	Literature Review	To analyze autologous dental bone graft (AUTO-BG), including its preparation process, clinical uses and applications in the mandibular region, as well as the limitations related to this technique.	-	Does not present results.	AUTO-BG is a new autogenous bone graft with osteoinductive, osteoconductive, and progressive replacement properties. It is useful in various clinical situations by offering biocompatibility without an immune response. Its procurement, need for carving, limited quantity, and lack of long-term data limit its routine use. Although the extent of the defect must be considered, it is a feasible option due to its origin and favorable results.
Brudnicki A. et al. [27]	2020	*The Cleft and Palate Journal*	Retrospective study	To evaluate the effect of the timing of secondary alveolar bone grafting (SABG) on craniofacial morphology in patients with complete unilateral cleft lip and palate (UCLP).	Descriptive statistics and comparative analysis	Regression models showed a limited effect of SABG on craniofacial morphology. Cephalometric variables between SABG and non-SABG groups showed no association with time when controlling for age of repair, cephalometric evaluation, cleft side, gender, and operator. Only maxillary length (Condylion-point A) was affected, with a 1-year delay in SABG, and was associated with a 0.52 mm increase in Co-point A distance.	SABG performed before the age of 8 years might have a limited negative effect on craniofacial morphology. However, the results must be confirmed by specialized centers that perform alternative surgical repairs.
Khan M. et al. [39]	2013	*Canadian Journal of Plastic Surgery*	Cross-sectional descriptive study	To review the Kernahan ’Y’ classification modified by Smith of cleft lip and palate deformities and describe the different anatomical subtypes.	In vivo	A total of 163 cases of lip and palate deformities were studied. Smith’s modified Kernahan ‘Y’ classification completely described the deformities in 93.9% of patients. However, it did not describe the different anatomical subtypes of submucosal cleft palate in 6.13% of patients.	The Kernahan ‘Y’ classification, modified by Smith and presented in this study, is capable of describing all variants of cleft lip and palate deformities, including the various types of submucous palate deformities.
McCrary M. et al. [25]	2021	*Oral and Maxillofacial Surgery Clinics of North America*	Literature Review	To review the pre-operative evaluation of an alveolar cleft, surgical treatment (including various grafting materials and methods), and post-operative care.	-	**Pre-operative evaluation:** Those who underwent treatment. Orthodontic treatment showed an improvement in bone volume compared to those who underwent bone grafting alone. **Surgical treatment:** It has been proven that autogenous grafts have greater effectiveness compared to others. The counterpart is the morbidity of the donor area. **Post-operative care:** Most frequent complications include wound dehiscence, infection, and graft resorption. Pain control, antibiotics, chlorhexidine.	Iliac crest autograft is the reference standard for bone grafting in alveolar fissures despite donor site morbidity.
Seifeldin SA et al. [40]	2016	*The Saudi Dental Journal*	Literature Review	To define whether the reconstruction of the alveolar fissure is a controversial topic in the literature.	-	The review of the literature is not conclusive regarding the most favorable moment for alveolar bone grafting.	Secondary bone grafts are the treatment most widely accepted for surgical reconstruction.
Mossaad A. et al. [13]	2019	*Macedonian Journal of Medical Sciences*	Randomized clinical study	To compare different grafting techniques to treat alveolar cleft defects.	In Vivo	In 24 cases: —Group A treated with autogenous iliac crest bone. —Group B treated with HA nano calcium with collagen membrane. —Group C treated with tissue engineering method using bone marrow stem cell extract and PRF membrane. Using CT, the study compared pre- and post-operative bone density. Group C showed the best bone density results, followed by group B. Group A presented cases of bone resorption and the lowest density values, in addition to adverse effects such as pain and scars in the donor area.	Bone substitutes, such as nanocalcium hydroxyapatite and bone marrow stem cell extract, proved to be reliable methods for bone grafting compared to autogenous iliac crest.
Liang F. et al. [18]	2018	*The Journal of Craniofacial Surgery*	Literature Review	To guide surgeons toward a safe and informed use of biomaterials in alveolar cleft reconstruction.	-	Does not present results.	No large-scale controlled clinical trials have been conducted to evaluate alternatives to autologous bone grafts in patients with alveolar clefts. There is insufficient clinical evidence in humans to demonstrate equivalence to bone autografts, which remain the standard of care.
Jariwala SH et al. [41]	2015	*3D Printing and Additive Manufacturing*	Clinical Evaluative Study	To provide the 3DP community with a concise introduction to additive manufacturing (AM)-based bone tissue engineering, including processes and materials, and how bone regenerative medicine can be optimized by controlling scaffold characteristics, such as surface topography.	In Vitro and In Vivo Tests	3DP offers the possibility of printing bone replacement materials with controlled chemistry, shape, porosity, and topography, allowing the printing of personalized bone grafts adapted to the patient and the specific clinical condition.	3DP and other AM methods are promising technologies for developing artificial bone structures or scaffolds that are as osteogenic as autografts or allografts. Additionally, there is great potential to use medical imaging combined with computer modeling and design to develop artificial bone grafts tailored to each patient’s specific defect.
Francisco I. et al. [42]	2021	*Bioengineering Journal*	Literature Review	To evaluate the effectiveness of current approaches in the regeneration of bone defects in non-syndromic patients with cleft palate.	-	**Bone formation volume** between ICBG and BMP-2 was not statistically significant (*p* = 0.704) and was estimated at 0.08 mm^3^ to 0.22 mm^3^ (95% CI: 0.35 to 0.51 mm^3^). **% bone formation** between ICBG and BMP-2 was estimated and turned out to be not statistically significant (*p* = 0.184) and was estimated at 67.92% to 51.05% (95% CI: 32.13 to 167.97%). **Bone height** between ICBG and BMP-2 was estimated and was not statistically significant (*p* = 0.520) and was estimated at 5.13% to 9.97% (95% CI: 10.49 to 20.74%).	This general review indicates that both BMP-2 and bone autograft are valid options for the treatment of patients with oral clefts since no differences were observed between them in terms of volume, filling, and bone height. However, these findings should be analyzed with caution due to several methodological gaps in the quality of the original studies.
Martín del Campo M. et al. [23]	2019	*International Journal of Molecular Sciences*	Literature Review	To analyze studies that have reported on the use of advanced biomaterials and cellular therapies for the regeneration of cleft lip and palate.	-	Hydroxyapatite-β-tricalcium phosphate showed good results in an animal model of alveolar cleft, with new bone formation in 8 weeks. Polylactic acid (PLA) and polyglycolic acid (PGA) have been used to fabricate porous scaffolds to regenerate the palate. 3D printing made it possible to develop a personalized jaw-shaped bone substitute using polycaprolactone, HA, alginate, and stem cells. A peptide derived from folic acid promoted the expression of genes related to osteogenesis in mesenchymal stem cells.	Synthetic bone grafts promote new bone formation. The combined use of bioceramics, polymer biomaterials, compounds derived from folic acid, morphogens, stem cells, and 3D bioprinting techniques are promising alternatives for tissue regeneration in patients with cleft lip and palate.
Garcia BA et al. [43]	2019	*Journal of Craniofacial Surgery*	Clinical Report	To discuss the use of umbilical cord stem cells in primary gingivoperiostioplasty for the treatment of alveolar clefts and thus avoid the use of alveolar bone grafts in the future.	In Vivo	Does not present results.	The use of umbilical cord stem cells with gingivoperiostioplasty during primary cheiloplasty could prevent the need for secondary alveolar bone grafts in patients with clefts, but more prospective, controlled studies with adequate follow-up are required to confirm these initial findings.
Mazzetti MPV et al. [44]	2018	*Journal of Craniofacial Surgery*	Clinical Report	To determine how stem cells obtained from the umbilical cord, blood, and placenta impact the post-operative recovery process of patients with cleft lip and palate after reconstructive surgery.	In Vivo	There were no surgical complications in the Stem Cell Group, with good scar appearance, little inflammation and no dehiscence. The SCG presented a higher % classification (-) in soft tissue inflammatory process, transient hypertrophy scar, and fibrosis between hard and soft palate. According to the palate evaluation, there were no statistical differences between the groups (*p* = 0.471). The group with stem cell injection had fewer post-operative complications and fibrosis.	Incorporating stem cells into cleft lip and palate surgeries can improve recovery by reducing inflammation and facilitating healing compared to conventional methods. Furthermore, no negative effects were observed when using stem cells with standard surgical procedures, so a protocol for the safe use of a high volume of stem cells was developed, establishing a basis for future research. Although more studies are required, current data are promising for the use of stem cells in surgery.
Khojasteh A. et al. [45]	2017	*Hindawi BioMed Research International*	Phase I Clinical Trial	To determine the efficacy of lateral branch cortical plating with buccal fat pad-derived mesenchymal stem cells (BFSCs) in the treatment of alveolar cleft defects.	In Vivo	**AIC Group**: Less new bone formation (70 ± 10.40%). **LRCP + BFSC group**: Closure of the defect and greater new bone formation (75 ± 3.5%) but less than the AIC + BFSC group. **AIC + BFSC Group**: The greatest amount of new bone formed (82.5 ± 6.45%).	The mixture of mesenchymal stem cells with bone from the anterior iliac crest could enhance bone regeneration in areas affected by alveolar fissures. To protect and sustain regenerative structures loaded with MSC, intraoral sources such as CPRL can be used. However, to advance the application of tissue engineering, the need for additional research with more participants and the evaluation of different combinations of cells, structural supports, and growth factors is suggested.
Shawky H. et al. [20]	2016	*The Cleft Palate-Craniofacial Journal*	Randomized clinical study	To evaluate the effect of platelet-rich fibrin (PRF) on the quality and quantity of bone formation in unilateral maxillary alveolar fissure reconstruction.	In Vivo	**Group A**: patients grafted with PRF + autogenous anterior iliac crest. **Group B**: patients grafted with autogenous bone graft only (control group). **Bone formation percentage:** Group A: between 79.74% and 88.4%, with an average % of 82.6% ± 3.9%. Group B: between 60.3% and 76.4%, with an average % of 68.38% ± 6.67%. There was a statistically significant increase in the % of newly formed bone in group A. The mean bone density (quality) of the newly formed bone was lower in group A than in group B, but the difference was not statistically significant.	The use of PRF in combination with autogenous bone was beneficial in improving the volume of newly formed bone. However, there is not enough evidence of improvement in bone density.
Silva Gomes Ferreira PH et al. [46]	2018	*Annals of Maxillofacial Surgery*	Literature Review	To evaluate and compare the different biomaterials used in surgeries for the closure of palatal and alveolar fissures as alternatives to autograft.	-	Imaging studies showed similar post-operative bone densities with autologous iliac crest graft or DBB between Groups I and II (*p* > 0.05). No differences in post-operative bone formation or resorption were observed between the autologous grafts associated with β-TCP (*p* = 0.306). The isolated use of bovine HA produced a density similar to the non-operated bone of the contralateral unfissured area (*p* = 0.328).	The use of autogenous bone in combination with demineralized bovine bone (DBB) or beta-tricalcium phosphate (β-TCP) can significantly reduce the amount of bone harvested from the iliac crest, as well as decrease patient morbidity and hospitalization time. On the other hand, the exclusive use of hydroxyapatite of bovine origin offers bone densities lower than those of bone itself. Although bone tissue engineering appears as an advanced option for alveolar grafts, further research is necessary for its practical application.
Green MA et al. [47]	2021	*Journal of Oral and Maxillofacial Surgery*	Retrospective Study	To determine if factors such as gender, type of fissure, implant site, number of implants, age for alveolar bone graft and implant, time between graft and implant, previous maxillary expansion, and Le Fort I osteotomy are associated with needing an additional bone graft before implantation.	Descriptive statistics and comparative analysis	A total of 84 implants were placed in 59 patients (64.2% women) who had undergone an alveolar bone graft, of whom 57.1% (n = 48) required an additional graft before placement. The mean time from alveolar bone graft to implant placement was significantly longer in patients who required supplemental grafting (8.1 vs. 5.4 years, *p* < 0.001). Patients who required supplemental bone were significantly younger at the time of alveolar bone grafting (10.1 vs. 12.3 years, *p* < 0.001). The cleft areas of patients who underwent a Le Fort I prior to implant placement required bone augmentation more frequently than patients who did not undergo a Le Fort I (58.7% vs. 33.3%, *p* = 0.03).	Additional bone grafts are often required before placing implants in canine pre-eruption cleft sites. It is recommended to perform them during Le Fort I osteotomy in patients with maxillary hypoplasia. Future research will evaluate bone defects and compare grafting techniques.
López-Cedrún JL et al. [48]	2014	*Journal of Cranio-Maxillo-Facial Surgery*	Retrospective Study	To describe a modified technique of the classic flap design in order to minimize injury to the dental papillae and periodontium of the dentition involved.	In Vivo	The study highlights excellent wound healing in most cases. Only a small percentage of patients faced minor complications such as dehiscence, oronasal fistula recurrence, and bone loss, but these did not significantly compromise clinical outcomes. Complications were generally manageable without the need for additional surgical interventions.	Keratinized gingival mucoperiosteal flaps facilitate effective and tension-free closure of the graft, thanks to their excellent vascularization and adequate mobility, also without the need for vertical incisions. This approach preserves the anatomy of the buccal sulcus and interdental papillae, ensuring a healthy periodontium that facilitates normal tooth eruption and successful orthodontic treatment in the areas near the cleft.
Rakhmatia YD et al. [49]	2013	*Journal of Prosthodontic Research*	Literature Review	To discuss and summarize the utility of various resorbable and non-resorbable membranes available in preclinical studies for guided bone regeneration (GBR), with a particular focus on titanium mesh membranes.	-	Membranes must meet 5 criteria: biocompatibility, ability to create space, occlusivity, tissue integration, and clinical manageability. Each type of membrane has advantages and disadvantages and these offer promising solutions in animal models, but an ideal membrane for clinical applications has not yet been established. Titanium mesh membranes offer excellent mechanical properties for GBR treatment.	Despite advances, an ideal membrane for clinical applications has not been established, so understanding the benefits and limitations of various materials will be of great value and assist in the selection of an optimal membrane.
Urban IA et al. [50]	2019	*Oral and Maxillofacial Surgery Clinics of North America*	Literature Review	To provide an updated synthesis of the evidence and practical guidance on the clinical use of guided bone regeneration (GBR) for alveolar bone reconstruction.	-	Does not present results.	GBR represents a plausible, viable, and effective alternative for the reconstruction of atrophic ridges. Crucial technical aspects, such as tension-free flap closure and stability of the graft and barrier membrane, are of vital importance to ensure the success of the intervention. The procedure requires extensive technical expertise and is indicated for patients with a low risk profile (i.e., with adequate oral hygiene measures and non-smokers).
Scheuermann M. et al. [51]	2019	*Journal of Oral and Maxillofacial Surgery*	Literature Review	To review the existing literature on spontaneous bone regeneration after surgical closure of clefts of the hard palate and to analyze the current evidence on this phenomenon.	-	Due to differences in patient characteristics and evaluation methods, it was difficult to compare the different surgical procedures. The use of mucoperiosteal flaps and adequate mucosal closure favor bone regeneration, which is greater in the central area of the palate. Complete closure could affect maxillary growth, but more studies are required to confirm this. Age is the only factor with possible relevance to the success of regeneration, according to current evidence.	Only a few studies with small sample sizes have been published on bone regeneration in the hard palate. More research is needed to define the best technique.
Pucciarelli MGR et al. [52]	2019	*Journal of Prosthetic Dentistry*	Clinical Report	To present a treatment guide for implant placement in patients with cleft lip and palate based on clinical cases and literature review.	In Vivo	Does not present results.	The treatment with implants in an area of congenital alveolar defect can be successful and improve function and aesthetics in patients with cleft palate. The importance of adequate diagnostic, surgical, and prosthodontic planning with long-term follow-up in these complex cases is emphasized.
Omara M. et al. [53]	2023	*Clinical Oral Investigations*	Randomized clinical study	To compare the effectiveness of mineralized plasma matrix (MPM) versus cancellous bone particles harvested from the anterior iliac crest in secondary alveolar grafts.	In vivo	There were no significant differences between the fissure volumes measured in both groups before surgery. After 6 months, the mineralized plasma matrix (MPM) group had significantly greater bone width and height than the control group.	The alveolar cleft graft with mineralized plasma matrix (MPM) showed greater stability in graft volume, labiopalatine bone width, and bone height compared to the control group treated with cancellous bone particles from the anterior iliac crest.

## Data Availability

The raw data supporting the conclusions of this article will be made available by the authors on request due to privacy reasons, as the dataset includes sensitive information derived from clinical studies involving pediatric patients.
